# Immune-related pancreatitis due to anti-PD-L1 therapy in a patient with non–small cell lung cancer: A case report

**DOI:** 10.1097/MD.0000000000029612

**Published:** 2022-07-22

**Authors:** Julie Malet, Boutheina Melki, Stéphane Chouabe, Gaëtan Deslée

**Affiliations:** a Department of Respiratory Diseases, Reims University Hospital, Reims, France; b Department of Respiratory Diseases, Charleville-Mézières Hospital, France; c Department of Pulmonary Pathologies and Cellular Plasticity, Inserm UMR-S 1250, Reims, France.

**Keywords:** case report, durvalumab, immune-related pancreatitis, lung cancer

## Abstract

Despite clinical-proven benefits of immune checkpoint inhibitors (ICIs) on advanced lung cancer, rare but life-threatening immune-related adverse events (irAEs) have been reported. Pancreatitis is a rare irAE that can occur with any ICI.

**Patient concerns:**

A 53-year-old man with locally advanced non–small cell lung carcinoma was treated with radiochemotherapy and then durvalumab (anti–programmed cell death ligand 1 therapy). Twelve weeks after the beginning of ICI, he reported abdominal pain and anorexia. Blood test showed high level of lipase. Abdominal computed tomography revealed a swollen pancreas. These findings were confirmed by magnetic resonance cholangiopancreatography and biliopancreatic endoscopic ultrasonography.

**Diagnoses:**

Grade IV immune-related pancreatitis.

**Interventions:**

The patient was treated with corticosteroid therapy, resulting in clinical, radiological, and biological improvement.

**Outcomes:**

During the first month, corticosteroid therapy could not be decreased under 1 mg/kg/d because of symptoms recurrence and lipasemia rerising. Four months after this episode, the patient died from acute ischemia of the lower limbs while he was on <20 mg/d of corticosteroid.

**Lessons:**

To the best of our knowledge, immune-related pancreatitis has been reported only with anti–programmed cell death 1 or anti–cytotoxic T lymphocyte antigen 4 therapies but never with anti–programmed cell death ligand 1 therapy. It is important to report such rare cases to improve diagnosis and management of irAEs.

## 1. Introduction

Durvalumab is a high-affinity immunoglobulin G1 kappa monoclonal antibody that suppresses the binding of programmed cell death ligand 1 (PD-L1) to programmed cell death 1 (PD-1) and CD80 and restores consequently the antitumor activity of T lymphocytes. It can be prescribed in patients with unresectable stage III non–small cell lung carcinoma who did not progress after at least 2 cycles of platinum-based chemotherapy associated with radiation therapy. Results from the PACIFIC study (NCT02125461) demonstrated markedly longer overall survival in the durvalumab group compared to placebo (57% versus 43.5% at 3 years).^[1]^ Even if the safety profile of durvalumab is considered acceptable, some serious immune-related adverse events (irAEs) can occur. We present here the first case of pancreatitis due to durvalumab.

## 2. Case presentation

### 2.1. Patient information

A 53-year-old man, without any medical history, was diagnosed with a locally advanced squamous cell carcinoma at stage IIIb (T4N2M0), PD-L1 0%. He received concomitant radiochemotherapy, including cisplatin and vinorelbine (4 cycles), and mediastinal radiotherapy (66 Gy). The disease was stable according to response evaluation criteria in solid tumors 1.1 criteria. Durvalumab was therefore prescribed (10 mg/kg every 2 weeks) as part of a temporary use authorization cohort.

According to the Jardé law in France, informed consent for publication was waived based on the retrospective noninterventional design and anonymous management of the patients’ data and was approved by the French national commission for personal data protection (CNIL, Comité National de l’Information et des Libertés).

### 2.2. Clinical findings and timeline

Twelve weeks after starting durvalumab, the patient was hospitalized for epigastralgia, anorexia, and weight loss. Physical examination showed serious dehydration, signs of malnutrition, and rebound tenderness.

### 2.3. Diagnostic assessment

**Figure 1.**
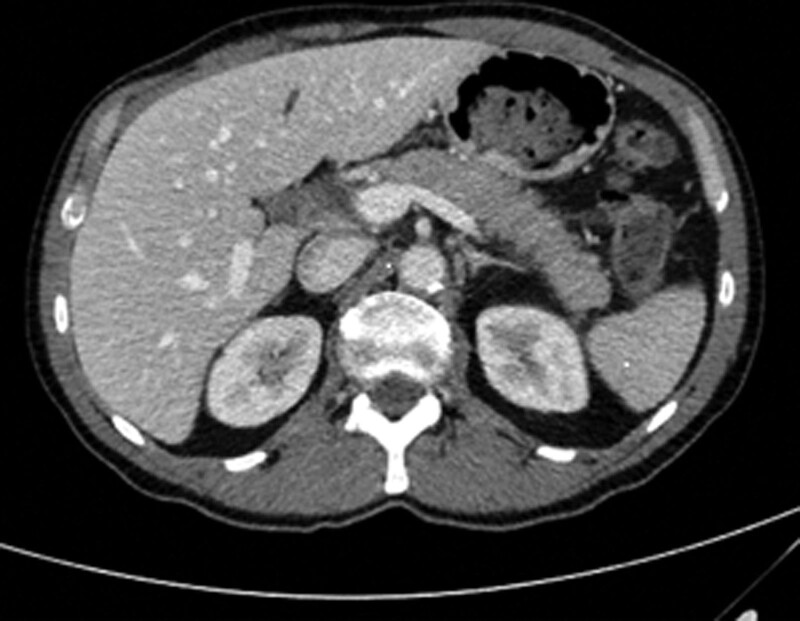
Abdominal CT scan showing swollen pancreas. CT = computed tomography.

Blood test showed more than ten times the normal level of lipase. Abdominal computed tomography scan revealed a swollen pancreas with homogeneous density and infiltration of peripancreatic fat, without any fluid collection nor necrosis (Stage C according to Balthazar score). There was no visible stone in the gallbladder (Fig. 1).

Transabdominal ultrasound did not find lithiasis or dilation of the bile ducts but an enlarged pancreas with some hypoechoic areas. Magnetic resonance cholangiopancreatography showed a dilatation in the lower part of the common bile duct measuring 16 mm, without dilation upstream. Biliopancreatic endoscopic ultrasonography did not find lithiasis or suspicious mass syndrome.

Other causes of acute pancreatitis, including gallstones, pancreatic tumors, pancreas divisum, alcoholism, and also lepidic, phosphocalcic, or other autoimmune etiologies, were ruled out by imaging and extensive blood tests. There was no clinical or biological argument for an infectious cause (viral, bacterial, mycotic, or parasitic). No mutations were found on molecular analysis of genes involved in hereditary and idiopathic pancreatitis (*PRSS1*, *SPINK1*, *CTRC*, *CPAI*, *CFTR*).

The diagnosis of immune-related pancreatitis, grade IV, occurring 12 weeks after the first administration of durvalumab was then confirmed.

### 2.4. Therapeutic intervention

Durvalumab was permanently discontinued. A strict fasting was implemented, associated with intravenous fluid hydration and analgesic treatment, without any benefit on clinical course after 7 days. Methylprednisolone was then administered at a dose of 1 mg/kg/d, leading to clinical improvement, decreased lipase level, and abdominal CT normalization after 2 weeks.

### 2.5. Follow-up and outcomes

Digestive symptoms and rerise in lipasemia reappeared 10 days after the decrease in methylprednisolone at 0.75 mg/kg/d, requiring to maintain 1-month methylprednisolone at a high dose (1 mg/kg/d). Methylprednisolone was tapered over a period of 3 months up to 20 mg/d without recurrence of symptoms. The patient died from acute ischemia of the lower limbs 4 months after his acute pancreatitis while he was on <20 mg/d of corticosteroid.

## 3. Discussion

According to Porcu et al,^[2]^ immune checkpoint inhibitor (ICI)–induced pancreatic injury is rare, occurring in up to 4% of the patients, and more often presenting as an asymptomatic lipase elevation or mildly symptomatic pancreatitis. Typical acute pancreatitis was reported in 30% of these patients. Among them, some patients may develop long-term damage, including chronic pancreatitis, type 1 diabetes, or exocrine pancreatic insufficiency. Our case illustrates a typical form of acute ICI-induced pancreatitis.

To the best of our knowledge, we did not find any reported case of acute pancreatitis related to durvalumab or anti-PD-L1 therapy.^[2,3]^ In PACIFIC trials, irAEs (all grades) are reported in 24.2% of the patients in the durvalumab group.^[1]^ According to a meta-analysis published in 2016, the incidence of acute pancreatitis associated with anti-PD-1 was 1.8%.^[4]^ In a systematic review and meta-analysis,^[3]^ Su et al underline the rarity of acute pancreatitis associated with different ICI (cytotoxic T lymphocyte antigen 4 [CTLA-4]: 0.9%–3%, PD-1: 0.5%–1.6%, CTLA-4 + PD-1: 2%–2.1%). Interestingly, this meta-analysis demonstrated that both CTLA-4 inhibitors alone and combination treatment of PD-1 could increase the risk of amylase or lipase elevation but not significantly increase the risk of pancreatitis.

The pathophysiology of immune-related pancreatitis associated with ICI, and specifically with the anti-PD-L1 durvalumab in our case report remains to be elucidated. Of note, CD8 + tissue-resident memory T cells in the pancreas (TRMs) express high levels of PD-1 whereas PD-L1 is mainly expressed by macrophages, which regulate pancreatic TRMs homeostasis.^[5]^ We can then hypothesize that durvalumab may interact in the cross-talk between macrophages and TRMs in the pancreas, leading to a local immune dysregulation in the pancreas. Furthermore, among ICI, durvalumab affects the immune response at a later stage compared to anti-CTLA-4 and anti-PD-1, which may explain the rarity of anti-PD-L1 related pancreatitis.

In our case report, the management of this durvalumab-related pancreatitis was conducted according to the American recommendations of the National Comprehensive Cancer Network for the management of ICI-related toxicities. The first line of care is similar to common acute pancreatitis (hospitalization, hydration, pain relief). The other aspects of management depend on the severity; in case of severe pancreatitis (grade 3–4), ICI must be definitively discontinued and prednisone (1–2 mg/kg/d) should be prescribed. Clinicians should pay attention while tapering corticosteroids, with symptoms that can reoccur at lower doses as described in our case. Furthermore, nonresponse to corticosteroid therapy can also occur.

## 4. Conclusion

To our knowledge, this is the first case report of acute pancreatitis due to durvalumab. Our case report should promote vigilance and notification by physicians of other cases of durvalumab-associated pancreatitis. Such rare cases are important to report to improve irAEs diagnosis and management.

## Author contributions

Conceptualization: Julie Malet, Boutheina Melki, Stéphane Chouabe, Gaëtan Deslée.

Validation: Julie Malet, Boutheina Melki, Stéphane Chouabe, Gaëtan Deslée.

Writing – original draft: Julie Malet, Boutheina Melki, Stéphane Chouabe, Gaëtan Deslée.

Writing – review & editing: Julie Malet, Boutheina Melki, Stéphane Chouabe, Gaëtan Deslée.
